# Effect of confinement of horse heart cytochrome c and formate dehydrogenase from *Candida boidinii* on mesoporous carbons on their catalytic activity

**DOI:** 10.1007/s00449-021-02553-3

**Published:** 2021-04-03

**Authors:** Naiara Hernández-Ibáñez, Vicente Montiel, Alicia Gomis-Berenguer, Conchi Ania, Jesús Iniesta

**Affiliations:** 1 Physical Chemistry Department and Institute of Electrochemistry, University of Alicante, 03080 Alicante, Spain; 2 INCAR, CSIC, Apdo 26, 33011 Oviedo, Spain; 3 CEMHTI, CNRS (UPR 3079) University of Orléans, 45071 Orléans, France; 4 Present Address: Department of Chemistry, School of Engineering Science in Chemistry, Biochemistry and Health, Royal Institute of Technology, KTH, Teknikringen 30, SE-100 44 Stockholm, Sweden

**Keywords:** Mesoporous carbon, Protein nanoconfinement, Cytochrome c, Formate dehydrogenase hydrogen peroxide, Carbon dioxide

## Abstract

This study reports the immobilization of two biocatalysts (e.g., cytochrome c—Cyt c—and the non-metalloenzyme formate dehydrogenase from *Candida boidinii*–cbFDH) on a series of mesoporous carbons with controlled pore sizes. The catalytic activity of the nanoconfined proteins was correlated with the pore size distribution of the carbon materials used as supports. The electrochemical behaviour of nanoconfined Cyt c showed direct electron transfer electroactivity in pore sizes matching tightly the protein dimension. The pseudo-peroxidase activity towards H_2_O_2_ reduction was enhanced at pH 4.0, due to the protein conformational changes. For cbFDH, the reduction of CO_2_ towards formic acid was evaluated for the nanoconfined protein, in the presence of nicotinamide adenine dinucleotide (NADH). The carbons displayed different cbFDH uptake capacity, governed by the dimensions of the main mesopore cavities and their accessibility through narrow pore necks. The catalytic activity of nanoconfined cbFDH was largely improved, compared to its performance in free solution. Regardless of the carbon support used, the production of formic acid was higher upon immobilization with lower nominal cbFDH:NADH ratios.

**Supplementary Information:**

The online version contains supplementary material available at 10.1007/s00449-021-02553-3.

## Introduction

The immobilization of biocatalysts (e.g., enzymes) can offer an economic improvement in many different fields, such as drinking water production and wastewater remediation [1], biomedical applications [2], (bio)sensing devices manufacture for analytical purposes [3], energy conversion in biofuel cells [4–6] and energy storage in bio-supercapacitors [7]. The immobilized biocatalysts (should) still display high specificity and selectivity, while overcoming some operational drawbacks related to protein aggregation and enhancing robustness under extreme conditions (viz*.* pH and temperature changes) that can remarkably modify the biochemical function and/or the structure of the non-immobilized biocatalysts. In this regard, the great recent advances in the use of heterogeneous biocatalysts for industrial applications [8] are mostly due to the development of efficient immobilization methodologies. However, there are some challenges to face, such as desorption and lixiviation, optimum enzyme orientation [9, 10], or cost reduction; viz. the biocatalysts should be easily handled and reusable, without any detrimental stability loss against moderate or hard reaction conditions, as well as showing enhanced electron transfer (ET) between the redox-active centers of the biocatalyst through the electrode substrate.

Confinement of proteins on suitable nanoporous electrode materials is an attractive approach for successfully ensuring an efficient and stable immobilization. Hence, a plethora of porous materials has been investigated for this purpose, including synthetic gels [11–13], conductive polymeric materials [14, 15] or carbon materials [16] as representative examples. Among them, nanoporous carbons are particularly interesting for this application, with plenty of studies reporting the enhanced stability of the nanoconfined biomolecules (e.g., glucose oxidase [17], acetylcholinesterase [18], bovine serum albumin [19], cytochrome c [20], dehydrogenases and oxidases [21] formate dehydrogenase [22, 23]) inside the pores for a variety of carbons. Such enhancement has been attributed to the characteristics of the carbons, including large specific surface areas, thermal and mechanical stability, and low cost [18, 20, 21, 24, 25]. However, an important challenge is to assure the long-term bioactivity retention of the immobilized molecule. For this, the choice of the carbon support (e.g., textural features, composition, conductivity) is important to control the carbon/biocatalyst interactions inside the pore to keep the biochemical function and enable high electron transfer rates and improved stability of the confined biomolecule.

The objective of this work was to explore the immobilization of two biomolecules of wide applicability and different molecular dimensions on mesoporous carbons with controlled mesoporosity, to evaluate the impact on their performance towards hydrogen peroxide electrosensing and the reduction of CO_2_, respectively.

Horse heart Cyt c and FDH from *Cadida boidonii* (cbFDH) were selected as model proteins implicated in a plethora of biosensing applications [26] for Cyt c, and in the reverse interconversion of CO_2_/formic acid [27] for cbFDH. It is worth mentioning that Cyt c is a relatively small hemoprotein widely used in superoxide anion sensing [28] or hydrogen peroxide biosensing [29]. At converse, cbvFDH is a bulky non-metalloenzyme that catalyses the reduction of CO_2_ in formic acid [22]. cbFDH typically displays a limited catalytic activity in solution, so a successful heterogeneous biocatalyst based on the immobilized enzyme would be of economic and technical interest for the manufacturing of biocathodes for the electrosynthesis of formic acid [30, 31].

To attain this goal, a series of mesoporous carbons combining large specific surface areas with specifically designed mesopores were used as electrodes and catalyst supports. The immobilization of both proteins was performed in neutral pH solutions, and the protein depletion was monitored by spectrophotometric techniques. The pseudo-peroxidase activity for the electrocatalytic reduction of H_2_O_2_ on the one hand, and the biotransformation of CO_2_ to formic acid on the other was investigated on the soluble and immobilized proteins. The novelty of the work resides on correlating the enzymatic activity retention of the biocatalysts with the confinement state and the pore dimensions. This approach is essential in the development of electrodes in sensing, catalysis, drug delivery, and biofuel cells.

## Materials and methods

### Chemical and reagents

Horse heart cytochrome c (Cyt c, purity ≥ 95%, lyophilized protein mainly in the oxidized form, molecular weight: 12,500 Da), formate dehydrogenase (FDH) from *Candida boidinii* (lyophilized powder, 5.0–15.0 units/mg protein of enzymatic activity according to the supplier) (from Sigma-Aldrich, Spain); β-Nicotinamide adenine dinucleotide sodium salt (NAD^+^ with purity ≥ 97%) and β-Nicotinamide adenine dinucleotide reduced dipotassium salt (NADH with purity ≥ 97%) were all purchased from Sigma-Aldrich. Nafion perfluorinated resin 5 wt% in isopropyl alcohol/water solution was purchased from Sigma Aldrich. The rest of chemicals used in this work were of the highest purity available. All solutions were prepared using double distilled water with a resistivity not less than 18.2 MΩ cm. Unless stated otherwise, protein solutions were prepared either in 0.10 M or 0.01 M sodium phosphate buffer solution (PB), pH 7.4, using Na_2_HPO_4_ and NaH_2_PO_4_ salts (from Sigma-Aldrich) and stored at 4 °C.

### Synthesis of the mesoporous carbons

A series of mesoporous carbons with different porous features was synthesized by the sol–gel polymerization of resorcinol and formaldehyde in water using Na_2_CO_3_ as catalyst, as reported elsewhere [32]. Briefly, the precursors were mixed at fixed molar ratios (resorcinol/water 0.06; resorcinol/formaldehyde 0.5; resorcinol/catalyst 100 and 200) under magnetic stirring and heated in sealed glass vessels at 95 °C for 4 h in an oven. Afterwards, the obtained wet polymeric resins were dried at 150 °C for 12 h and then carbonized at 800 °C (i.e., 100 mL/min N_2_, 1 h). The mesoporous carbons were labeled as Gx, where x refers to the resorcinol/catalyst molar ratio (i.e., G100 and G200). A mesoporous carbon modified with ca. 5 wt% of carbon black (Super P, TIMCAL) was prepared by incorporating the additive with the reactants [33]. The nomenclature of this sample was G200CB. Another mesoporous carbon was obtained by chemical activation in K_2_CO_3_ of the resin prepared using a resorcinol/catalyst molar ratio of 200 (ca. resin:K_2_CO_3_ ratio of 1:1, heating at 800 °C, 300 mL/min N_2_, 1 h) [34]. The nomenclature of the chemically activated sample was G200K. All the prepared samples were ground and sieved using a cut-off mesh of 75 microns, to reduce the average particle size before the preparation of the electrodes (SEM images of selected samples are shown in the Electronic Supplementary Information-ESI-file, Fig. ESI-1).

### Textural characterization

The porosity of the mesoporous carbons was analyzed by high-resolution equilibrium N_2_ adsorption–desorption isotherms at − 196 °C, recorded in a volumetric analyzer (Micromeritics). Before the analysis, the samples were degassed under dynamic vacuum at 120 °C for 17 h. Analysis conditions were programmed to ensure equilibrium data (e.g., the average elapsed time for each isotherm was 90–120 h). Ultrahigh purity (i.e., 99.995%) nitrogen gas was supplied by Air Products. The isotherms were used to calculate the specific surface area using the Brunauer–Emmett–Teller theory (*S*_BET_), and the total pore volume (*V*_TOTAL PORES_). The pore size distribution (PSD) analysis in the full micro–mesopore range was calculated using the two-dimensional non-linear density functional theory model (2D-NLDFT-HS) assuming pore surface heterogeneity [35].

### Preparation of the inks and electrodes

For the evaluation of the amount immobilized on the carbons, solutions of Cyt c and cbFDH were prepared in 10 mM PB at pH 7.4 with an initial concentration of 0.4 and 0.5 mg/mL, respectively. The uptake of both biomolecules is expressed per gram (e.g., mg cbFDH/mg Gx) and area (e.g., mg cbFDH/m^2^ Gx) of carbons.

Before immobilization, the enzymatic activity of cbFDH (control experiment) was verified, obtaining a value of 5.8 U/mg. All the activity measurements of cbFDH were carried out using the same enzyme batch.

For the immobilization of the proteins, solutions of 5.0 mg/mL of Cyt c and 0.5 mg/mL of cbFDH were set as initial concentration. Adequate amounts of carbon materials were added to the solutions of the biomolecules to obtain a carbon suspension of 1.0 mg/mL; the suspensions were stirred during 6 days at 4 °C in closed vials under argon atmosphere. Thereafter, the suspensions were centrifuged at 1300 rpm for 5 min, and the supernatant solution was removed. The protein-containing carbons were rinsed several times with PB to remove the weakly bounded fractions of Cyt c and cbFDH. The amount of protein immobilized was calculated from the mass balance of the amount remaining in solution detected by UV–Vis spectrophotometry (ca. 411 nm—Soret region—for Cyt c; 280 nm for cbFDH). After immobilization, the solids were resuspended in 1.0 mL of 0.1 M PB pH 7.4, and then stored at 4 °C under argon atmosphere until further use.

For the preparation of the electrodes, the above-prepared protein-containing carbon suspensions were first sonicated for 15 min (Ultrasons P selecta) and then an adequate volume of 5 wt% nafion aqueous solution was added to reach a final concentration of ca. 20 wt% nafion vs the weight of solid residue. The mixture was sonicated for 40 min, maintaining the temperature lower than 30 °C. Then, an accurate volume of 20 μL of the ink was drop-casted onto a glassy carbon electrode surface (GCE, 3.0 mm diameter, from Goodfellow, United Kingdom) and then dried under nitrogen atmosphere. The GCE was previously polished using alumina (ca. 1.0, 0.3 and 0.05 μm) water suspensions. The electrode films were labelled as Cyt-c/Gx/GCE, where Gx refers to the mesoporous carbon (*vide supra*). For comparison purposes, the corresponding control electrodes prepared in the absence of protein (films labelled as Gx/GCE) were also measured.

### *Electrochemical measurements and H*_*2*_*O*_*2*_* detection*

Cyclic voltammetry (CV) and chronoamperometry measurements were performed with a potentiostat/galvanostat system Autolab PGSTAT X (Eco Chemie, the Netherlands) and controlled by Autolab GPES software version 4.9 for Windows XP. Experiments were performed in a three-electrode electrochemical glass cell, using a gold wire as counter electrode, a AgCl/Ag (1.0 M KCl) as reference electrode, and the prepared working electrodes (either Cyt-c/Gx/GCE or Gx/GCE). Due to the nanoporous nature of the carbon materials, before any electrochemical measurement, the electrodes were immersed in 0.1 M PB for at least 30 min at open circuit voltage, to guarantee wettability of the porosity of the electrodes. Chronoamperometry (CA) experiments were performed at − 0.5 V versus the reference electrode for exploring the pseudo-peroxidase activity of Cyt-c/Gx/GCE. All CA experiments were performed under stirring conditions by adding consecutive aliquots of 10 mM H_2_O_2_ aqueous solution into the electrochemical cell containing either 0.1 M PB at pH 7.4 or 0.1 M NaH_2_PO_4_ adjusted at pH 4.0. In the latter solution, a 0.1 M PB at pH 5.8 was adjusted by the addition of H_3_PO_4_. All electrochemical measurements were carried out at 293 ± 2 K under an argon atmosphere.

### Carbon dioxide enzymatic reduction

The enzymatic activity of cbFDH was performed spectrophotometrically recording the absorbance increment of NADH production at 340 nm during the oxidation of formic acid (FA) in the presence of a certain concentration of cbFDH and cofactor NAD+, following the activity assay procedure reported in [31]. The enzymatic activity of cbFDH immobilized in the mesoporous carbons was investigated by the chemical reduction of CO_2_ to render FA. For comparative purposes, the enzymatic reaction was also performed with cbFDH in solution. About 410 μL of 1.0 mM NADH in 0.1 M PB at pH 7.4 was added into 1.0 mL of either cbFDH in solution (from 0.35 to 5.32 mg cbFDH/mL) or a cbFDH/Gx suspensions (ca. 1.0 mg cbFDH/Gx), previously saturated with CO_2_. During the chemical reaction, a CO_2_ flow was continuously bubbled through the solution/carbon dispersion in 0.1 M PB with a flow rate of 100 cm^3^/min using a mass flowmeter (SIERRA instrument, inc. Smart-Track 2). The chemical reaction was carried out for 5 h at 293 ± 2 K. Thereafter, the cbFDH/Gx suspensions were centrifuged at 1300 rpm for 5 min, and then the supernatant was removed for further inspection of the FA concentration in solution using an ion chromatograph equipped with chemical suppression (An Cat, 850 detection Professional IC). Experiments were performed using Metrosep A Supp 7-250 column (Metrohm with 250 × 4.0 mm column dimensions and particle size of 5 µm). Mobile phase was 3.6 mmol/L sodium carbonate with a flow rate of 0.8 mL/min at 40 °C.

## Results and discussion

Cyt c is a hemoprotein with a molecular weight of ca. 12,500 Da and 104 amino acids, with spherical shape with circa 3 nm diameter [36]. This protein has an exposed heme group and co-axial lysine residues to the central Fe atom that can selectively interact with charged substrates in a similar reaction to that of peroxidases. On the other hand, cbFDH is a larger protein (homodimer) compared to Cyt c, with a molecular weight of ca. 81,209 Da in a homodimer configuration, having a unit cell *a*:*b*:*c* of 5.35, 6.85, 10.95 nm, [37]. Both proteins represent a good paradigm for exploring how the immobilization on a series of mesoporous carbons with varied pore architectures correlates with the direct electron transfer and electro-sensing of H_2_O_2_ in the case of Cyt c, and the enzymatic activity in terms of FA production rate in the case of cbFDH.

The characteristics of the mesoporous carbons (texture, composition) selected for this study have been described in our previous works [17, 38]. We herein introduce some physicochemical parameters for data interpretation (Table ESI-1, Figures ESI-2 and ESI-3), aiming at elucidating the role of the nanopore confinement on the activity of the immobilized proteins. Briefly, all the carbons displayed a well-developed pore structure in the full micro-mesopore range, with differences in the average mesopore size. In addition, it should be pointed out that all the carbons displayed basic character and hydrophobic nature, for which no specific interactions are expected with either of the proteins. This is important to isolate the effect of nanopore confinement of the immobilized enzyme on its electrochemical (pseudo)enzymatic activity from other (non-catalytic) contributions.

### Immobilization of Cyt c

Initially, the influence of the porous features of the studied mesoporous carbons on the immobilization of Cyt c was investigated by UV–Vis spectrophotometry for an initial concentration of 0.4 mg Cyt c/mL in 10 mM PB at pH 7.4, as aforementioned in the “Materials and methods” section. Table 1 shows the amount of protein adsorbed either per unit weight or per surface area of mesoporous carbon along with the overall protein adsorption at those conditions (weight percentage). For all the carbons with the exception of sample G200K, the adsorption of Cyt c was complete under experimental conditions used, as inferred from the absence of protein in the supernatant solutions. The complete depletion of the protein from the solution indicates that the amount immobilized is below the saturation capacity of the carbons. In addition, uptakes of 100% for these carbons also indicate that the pores are wide enough to accommodate the protein.

**Table 1:** Adsorption capacity of Cyt c in the studied mesoporous carbons (Gx), expressed per gram (mg Cyt c/mg Gx) and area (mg Cyt c/m^2^ Gx) of the carbon supports

Mesoporous carbon	mg_Cyt c_ (mg_Gx_)	mg_Cyt c_ (m^2^_Gx_)	wt% adsorbed
G100	0.35	0.40	99.9
G200	0.38	0.46	100.0
G200CB	0.38	0.58	100.0
G200K	0.13	0.10	37.4

Initial Cyt c concentration: 0.4 mg/mL

**Fig. 1:**
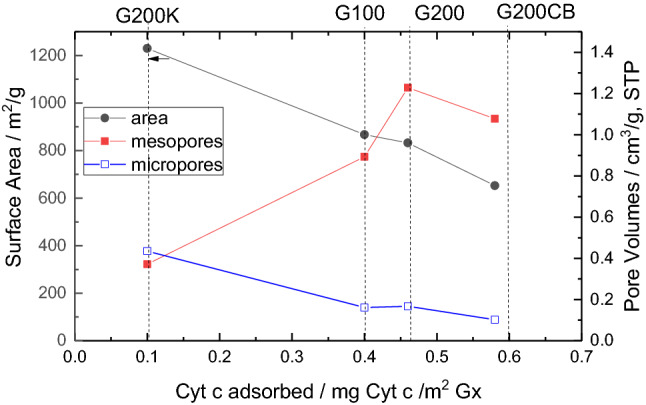
Correlation between the uptake of Cyt c and selected textural parameters of the studied carbons: surface area (left Y axis), and micropore and mesopore volumes (right Y axis), evaluated from the N_2_ adsorption isotherms at 77 K. Lines are guides for the eye to clarify the samples labelling

For carbon G200K, only ca. 37% of the overall amount of Cyt c was immobilized. These results are consistent with the analysis of the textural features of the samples (Table ESI-1, Figures ESI-2, ESI-3) and the dimensions of the protein according to literature (ca. 3 nm) [36]. Indeed, the differences in the microporosity of the samples are not expected to be relevant for the immobilization of Cyt c, which is expected to occur only in the mesopores, which size commensurate with the dimensions of the protein (ca. pore sizes equal or higher than their dimensions, *vide supra*). The correlation of Cyt c uptake with the mesoporosity rather than the microporosity (or surface area) of the carbons is shown in Fig. 1. It can be observed that the uptake was smaller for those carbons with a high contribution of microporosity to the overall porosity, whereas it followed an increasing trend with the volume of mesopores.

While all the carbons displayed multimodal PSDs with average mesopore sizes following the order: G100–G200K < G200 < G200CB (Figure ESI-3), the shape of the gas adsorption isotherms and hysteresis loops revealed important differences concerning the connectivity of the porous structure that affect the uptake of the studied protein. Samples G100, G200 and G200CB are characterized for rather uniform (meso)pores size distributions, with mesopores connected by wide pore necks (Figure ESI-3). At converse, carbon G200K presents constrictions in the porous network [39], with the mesopore cavities interconnected by narrow pore necks of ca. 3–4 nm; the dimensions of the pore necks are much smaller than those of G100, though interestingly both samples presented quite similar size of the main cavities. Hence, the lower adsorption capacity of Cyt c on carbon G200K (Table 1) compared to the other carbons of the series is attributed to (1) its lower volume of mesopores (account for a fraction of ca. 42%) and (2) the narrow dimensions of the pore necks that would hinder the accessibility of Cyt c to enter and accommodate in the main mesopores (6–7 nm). Since the immobilization was carried out for 6 days (see “Materials and methods” section), it is very unlikely that the overall uptake could be increased if longer adsorption times were allowed.

Finally, after the immobilization, the self-lixiviation (desorption) of the enzyme in the storage solution was evaluated; a negligible desorption of immobilized Cyt c was observed upon storage at 4 °C for up to five months for all the carbons, confirming the long-term stability of the immobilized protein.

The electrocatalytic activity of immobilized Cyt c was evaluated for the reduction of H_2_O_2_. Cytochromes are redox proteins that transfer electrons by oxidation and reduction processes via the heme group (iron bound to a porphyrin) (*vide supra*). Thus, during the negative scan in the cyclic voltammograms recorded on the prepared Cyt-c/Gx/GCE electrodes, Fe(III) can be reduced to Fe(II) and then oxidized back to Fe (III) during the reverse positive scan [40].

**Fig. 2:**
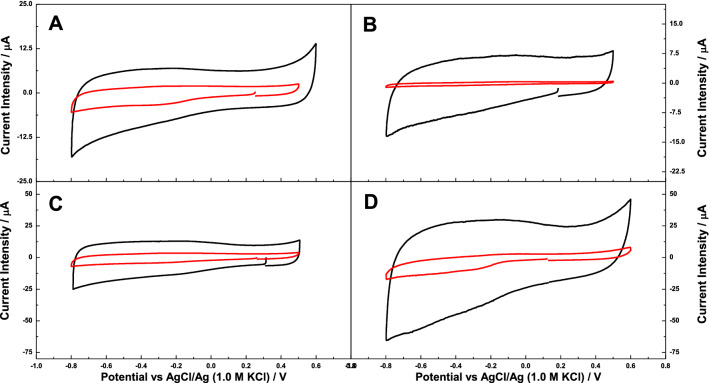
Cyclic voltammograms showing the electrochemical response of the pristine mesoporous carbon based electrodes (black lines), and after the immobilization of Cyt c (red lines) in 0.1 M PB 7.4. Scan rate: 0.010 V/s. Third scan recorded. **a** G100/GCE **b** G200/GCE **c** G200CB/GCE and **d** G200K/GCE. (Color figure online)

Figure 2 shows the cyclic voltammetries of the electrodes Cyt-c/Gx/GCE, prepared by immobilization of Cyt c on the mesoporous carbons; data corresponding to the series GCE/Gx in the absence of the enzyme are also shown for comparison purposes. For the control voltammograms in the absence of Cyt c, a relatively large contribution of the capacitance was observed, as expected due to the microporous character of the carbons (Table ESI-1) and hence the formation of the electrical double layer. Indeed, the largest capacitive contribution was observed for carbon G200K, in agreement with its higher surface area and micropore volume.

Interestingly, the immobilization of Cyt c resulted in a decrease in the contribution of the capacitive current of the voltammograms. This suggests that upon immobilization of Cyt c, the micropores would be partially blocked by the non-conductive protein, thus impeding the flux of ions through the porous network of the carbon material, overall resulting in a lower contribution of the double layer.

**Fig. 3:**
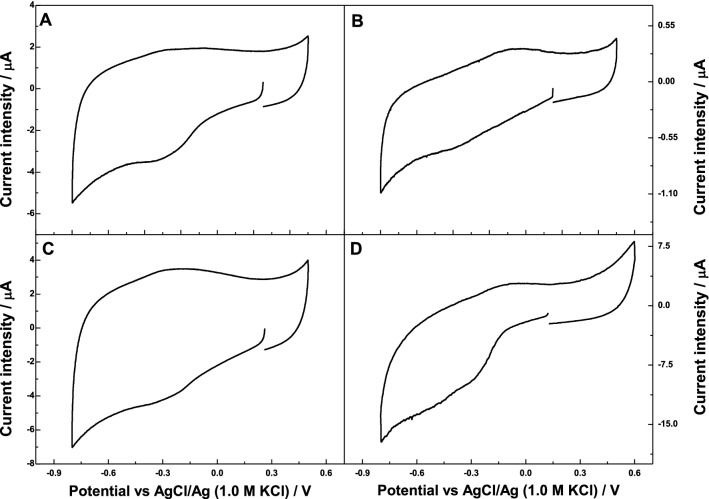
Cyclic voltammograms showing the electrochemical response of Cyt c immobilized on the mesoporous carbons based electrodes in 0.1 M PB 7.4. Scan rate: 0.010 V/s. First scan recorded. **a** Cyt-c/G100/GCE, **b** Cyt-c/G200/GCE, **c** Cyt-c/G200CB/GCE and **d** Cyt-c/G200K/GCE

A deeper look at the electrochemical behaviour of Cyt c confined in the mesoporous carbons is shown in Fig. 3. During negative scan, a cathodic shoulder is observed at around − 0.33, − 0.37, − 0.32 and − 0.31 V, along with an anodic shoulder in the reverse positive scan at − 0.280, − 0.050, − 0.220 and − 0.040 V, for the electrodes Cyt-c/G100/GCE, Cyt-c/G200/GCE, Cyt-c/G200CB/GCE, and Cyt-c/G200K/GCE, respectively. A shoulder-to-shoulder potential separation of over 270 and 320 mV was obtained for G200K and G200, respectively. This could be ascribed to a high irreversibility of the redox process of Cyt c after adsorption, indicating a sluggish direct electron transfer (DET) between the active centre of the protein and the carbon matrix. On the other hand, a shoulder-to-shoulder potential separation of 50 and 90 mV was obtained for Cyt-c/G100/GCE and Cyt-c/G200CB/GCE electrodes, respectively indicating the beneficial effect of immobilization on these carbons for achieving a fast DET. These results also correlate with the trend observed for reduction of the double layer formation after the immobilization of Cyt c on the mesoporous carbons. Thus, a slow DET could be correlated with a highest reduction of the specific capacity of both, Cyt-c/G200/GCE, and Cyt-c/G200K/GCE electrodes, as opposed to the immobilization on G100 and G200CB. Interestingly, the latter carbons displayed very different porous features with the smallest and highest average mesopore size, respectively (Figure ESI-2). In the case of carbon G100, the tight confinement of the protein in the mesopores of commensurate size might be responsible for this behaviour. In the case of G200CB, the enhancement of the electrochemical response might be attributed to the higher conductivity of this carbon material (ca. 200 mS/cm) compared to the series of carbons prepared without conductive additive (ca. 50–60 mS/cm) [33]. Additionally, the effect of scan rate was explored on all Cyt-c/Gx/GCE electrodes; however, the lower scan of 5 mV/s does not ameliorate the resolution of the shoulder-to-shoulder potential separation, whereas at higher scan rates, the cyclic voltammograms were mostly dominated by the capacitive process.

**Fig. 4:**
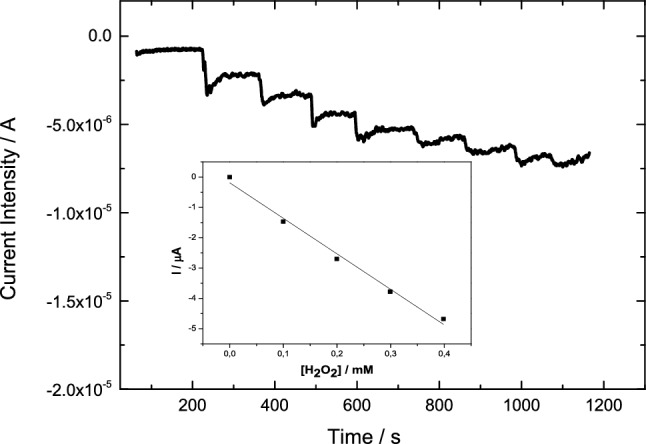
Chronoamperometric response at − 0.5 V for the electroreduction of H_2_O_2_ using Cyt-c/G200CB/GCE electrode over consecutive additions of H_2_O_2_ solution in 0.1 M PB at pH 7.4. Inset: calibration curve of the current intensity versus H_2_O_2_ concentration. Linear regression (*R*^2^) equals 0.996

The pseudo-peroxidase activity of nanoconfined Cyt c was examined on the electrodes Cyt-c/Gx/GCE; viz. chronoamperometries of the electrodes were carried out at a controlled potential of − 0.5 V and increasing concentrations of H_2_O_2_. Figure 4 shows a representative chronoamperometric response of Cyt-c/G200CB working electrode as an example; data corresponding to the rest of the mesoporous carbons are summarized in Table 2. It should be mentioned that in the absence of Cyt c (control experiments for electrodes Gx/GCE), negligible changes in the current intensity were observed after the successive additions of hydrogen peroxide, irrespectively of the pH. As an example, Figure ESI-4 depicts the chronoamperometric curves by comparing the transients of electrodes Cyt-c/G200/GCE and G200/GCE. This indicates that none of the studied mesoporous carbons presented electrocatalytic activity for the reduction of H_2_O_2_ under the applied conditions. By contrast, electrode Cyt-c/G200CB/GCE exhibited a remarkable increase in the current intensity, demonstrating the electrochemical reduction of H_2_O_2_ in PB, according to the following reactions [41]:

1 $${\text{Cyt - c - Fe}}\left( {{\text{III}}} \right) + {\text{e}}^{ - } \to {\text{cyt - c - Fe}}\left( {{\text{II}}} \right)$$

2 $$2{\text{ Cyt - c - Fe}}\left( {{\text{II}}} \right) + 2{\text{H}}^{ + } + {\text{H}}_{2} {\text{O}}_{2} \to 2{\text{ Cyt - c - Fe}}\left( {{\text{III}}} \right) + 2{\text{H}}_{2} {\text{O}}$$

**Table 2:** Analytical parameters obtained from the pseudo-peroxidase activity of the different Cyt-c/Gx/GCE electrodes in 0.1 M PB pH 7.4

Mesoporous carbon	Sensitivity (μA/mM/mg_Cyt c_)	LoD (μM)	Concentration range (μM)
G100	144.8	87	100–400
G200	70.9	11	300–700
G200CB	178.2	59	100–500
G200K	5.4 × 10^–5^	14	110–425

Limit of Detection (LoD) is calculated as three times the noise level

As seen, a typical steady-state current is obtained after the addiction of H_2_O_2_ to get a final concentration of 50 μM with a response time between 20 and 25 s; this is slightly higher than the 10 s obtained for the H_2_O_2_ detection using Cyt c immobilised on ordered macroporous carbons reported by Zhang [42], or the ca. 6 s using mesoporous carbon nanospheres in a 50 mM phosphate buffer reported by Wang et al. [43].

Table 2 shows the concentration ranges for the electroreduction of H_2_O_2_ as well as the limits of detection (LoD) obtained for all the studied Cyt-c/Gx/GCE electrodes. Electrodes Cyt-c/G100/GCE and Cyt-c/G200CB/GCE depicted a similar pseudo-peroxidase activity. Moreover, Cyt-c/G200/GCE electrode exhibited half the sensitivity of the electrodes based on either G100 and G200CB carbons. In contrast, almost no electrochemical response was obtained when using the Cyt-c/G200K-based electrode. This could be attributed to the low Cyt c loading of this carbon (Table 1), providing a low signal and/or to a sluggish electron communication between the active centre of the protein and the carbon material. These results are consistent with the electrochemical response of the electrodes observed in Figs. 2 and 3, with the systems Cyt-c/G200 and Cyt-c/G200K showing a slow DET and large irreversibility evidenced by the shoulders associated to the electron transfer reactions of Cyt c. Figure ESI-5A,B shows the correlation between the electrocatalytic activity of immobilized Cyt c and the dimensions of the mesopores (both pore body and neck) in the carbons. As seen, the highest sensitivity and lowest LoD were obtained for the carbons combining adequate dimensions of the main mesopore cavities and accessibility through big enough pore necks. Indeed, it can be observed that the higher conductivity of sample G200CB over the rest of the carbons would be responsible for the higher sensitivity of the electrode (due to lower current losses); however, this fact did not imply a better electrocatalytic performance in terms of LoD. This is most likely due to the large mesopore cavities of the carbon (exceeding the dimensions of Cyt c) and thus resulting in a non-optimizing pore confinement, demonstrating the key role of the latter.

To evaluate if the confinement in the mesopores affects the conformation state of Cyt c, the electrochemical response of Cyt-c/Gx/GCE electrodes was investigated in 0.1 M PB at pH 4.0. Indeed, pH is known to have a strong effect in the conformational changes in the heme group of Cyt c, with consequences on the electron transfer kinetic and therefore on the pseudo-peroxidise activity of the protein [40, 44–46]. Cyt c may exist in solution in five reversible pH-dependent conformational states: I, II, III, IV, and V with pKa values of 0.42, 2.50, 9.35, and 12.76, respectively [40, 47].

**Fig. 5:**
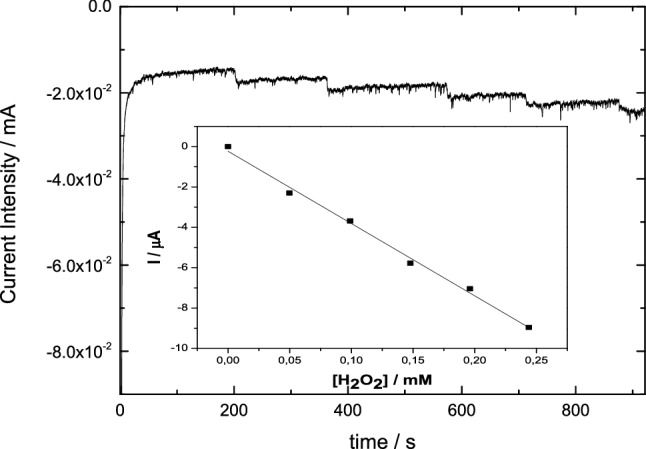
Chronoamperometric response at − 0.5 V for the electroreduction of H_2_O_2_ using Cyt-c/G200/GCE electrode over consecutive additions of H_2_O_2_ solution in 0.1 M NaH_2_PO_4_ adjusted at pH 4.0. Inset: calibration curve of the current intensity versus H_2_O_2_ concentration. Linear regression (*R*^2^) equals 0.997

Figure 5 shows the chronoamperometric response of Cyt-c/G200 at a controlled potential of − 0.5 V with increasing H_2_O_2_ concentration at pH 4.0. The electrocatalytic response followed a linear trend with the concentration of H_2_O_2_ in the range from 50 to 250 µM (inset of Fig. 5). The sensitivities obtained at pH 4 was improved for all the electrodes, compared to the measurements at pH 7.4 (Table 3). This finding is likely ascribed to the more adequate conformation of the protein at this pH, with a bigger exposition of the heme pocket [44, 45], thereby providing a higher pseudo-peroxidase activity of Cyt c. Compared to pH 7.4 (where Cyt c protein is in state III—His18 and amino acid residue Met80 in axial positions of the heme group—[44, 45], the response time was enhanced when the electrocatalytic activity of immobilized Cyt c was performed at pH 4.0. The sensitivities for the determination of H_2_O_2_ and LoD values at pH 4 correlate well with the dimensions of the main cavity and necks of the mesopore (Figure ESI-5C,D). In terms of LoD values, these were slightly improved at pH 4, reaching values close to those reported for other carbon materials [42, 43].

**Table 3:** Analytical parameters obtained from the pseudo peroxidase activity of the different Cyt-c/Gx/GCE electrodes in 0.1 M NaH_2_PO_4_ adjusted at pH 4.0

Mesoporous carbon	Sensitivity (μA/mM/mg_Cyt c_)	LoD (μM)	Concentration range (μM)
G100	1042.7 0.015^a^	47	50–300
G200	2012.9 0.038^a^	22	50–250
G200CB	799.8 0.010^a^	20	50–300
G200K	1814.1 0.10^a^	37	50–300

LoD is calculated as three times the noise level

^a^Sensitivity expressed in μA/μM for comparison purposes

### Immobilization of cbFDH

**Fig. 6:**
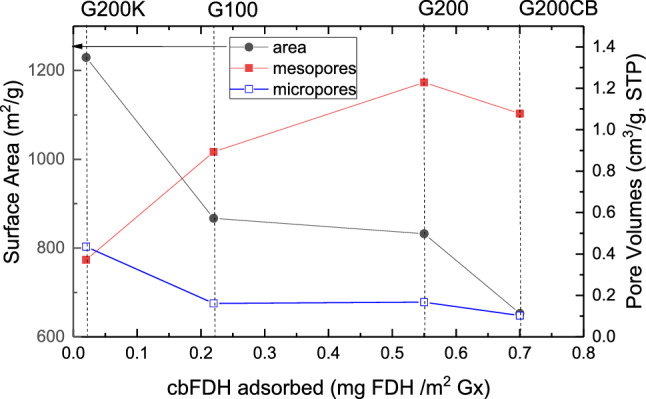
Correlation between the uptake of cbFDH and selected textural parameters of the studied carbons: surface area (left Y axis), and micropore and mesopore volumes (right Y axis). Lines are guides for the eye to clarify the samples labelling

Table 4 shows the amount of cbFDH adsorbed on the studied mesoporous carbon, expressed per mass and per surface area of carbon. As seen, the differences in the uptake of cbFDH are very pronounced among the carbons, with quite low adsorption capacities for samples G100 and G200K (ca. 41 and 5%, respectively). This is in agreement with the dimensions of cbFDH and the porous features of these carbons who displayed the smallest average mesopore size of the series (Figure ESI-3). This is more clearly illustrated in Fig. 6, showing the increasing trend of the uptake of the enzyme with the volume of mesopores and regardless the microporosity of the carbons.

**Table 4:** Adsorption capacity of cbFDH in the studied mesoporous carbons (Gx), expressed per gram (mg cbFDH/mg Gx) and surface area (mg cbFDH/m^2^ Gx) of the carbon supports. Initial cbFDH concentration: 0.5 mg/mL

Mesoporous carbon	mg_cbFDH_ /mg_Gx_	mg_cbFDH_/m^2^	wt% adsorbed
G100	0.19	0.22	41.2
G200	0.46	0.55	100.0
G200CB	0.46	0.70	100.0
G200K	0.02	0.02	5.2

The large differences in the cbFDH uptake of G100 and G200K (Table 4) contrast with the fact that the differences in the average distribution of mesopore size distributions are not so large; this points out to accessibility limitations of the enzyme to enter the mesopore cavities. Thus, for carbons G100 and G200K, where the main pores are connected through ca. 5 and 4 nm pore mouths, respectively (Figure ESI-3), cbFDH with a “rugby” shape (vide supra) would show some limitations to access the pores. In contrast, samples G200 and G200CB that presented wider mesopores connected through larger pore necks (ca. 8–9 nm) presented full uptake of the enzyme as they can accommodate better the enzyme and the access to the pores is not restricted. Regarding the long-term stability of immobilized cbFDH, the lixiviation tests confirmed the lack of desorption of the enzyme upon storage in the PB solution for five months.

The catalytic activity of the enzyme after the immobilization on the different carbons was checked through the reduction of CO_2_ to produce formic acid in a saturated CO_2_ solution. The reaction time was set at 5 h (reaction times generally range between 2 and 8 h for soluble and immobilized cbFDH [31]. The activity of the bare enzyme from solution was first determined varying the cbFDH-to-NADH ratio (mg_cbFDH_/µmol_NADH_) for the enzyme dissolved in 0.1 M PB at pH 7.4 under stirring (Figure ESI-6). The production rate of FA (µmol FA/min cbFDH) of soluble cbFDH followed a linear increasing response with the cbFDH-to-NADH ratio. It should be mentioned that the effects of the different hydrodynamic conditions on the reaction yield (e.g. stirring conditions, flow rate of CO_2_ through the reactor, formation of CO_2_ bubbles, local pH changes, temperature variation) have not been optimized and remained out of the scope of this study. In our case, we have used similar experimental conditions for immobilized and un-supported cbFDH, which allows the comparison of the obtained data disregarding other optimization parameters.

Table 5 compiles the results obtained for the immobilized cbFDH in the different mesoporous carbons for the same amount of NADH cofactor. The NADH/cbFDH ratio will vary for the different experiments due to the different loading of the enzyme in the carbons. First, it is worth noting that the immobilized cbFDH was still active for the enzymatic reduction of CO_2_ to FA for all the carbons (even for G200K, that presented a low enzyme uptake), with FA concentrations ranging between 9 and 12 ppm. This denotes that under our experimental conditions, FA concentration is almost independent of the cbFDH loading on the carbons.

**Table 5:** Production rates of formic acid (FA) using immobilized cbFDH on the mesoporous carbons resuspended in 0.1 M PB-pH 7.4 in the presence of CO_2_

FDH immobilized	G100	G200	G200CB	G200K
cbFDH immobilized (mg)	0.19	0.46	0.54	0.02
Ratio cbFDH/NADH (mg_cbFDH_/µmol_NADH_)	0.23	0.56	0.66	0.024
FA (ppm)	11.30	12.50	9.60	12.30
FA production rate (µmol FA/min)	1.2 × 10^–3^	1.3 × 10^–3^	1.0 × 10^–3^	1.3 × 10^–3^
FA production rate (µmol FA mg_cbFDH_/min)	6.1 × 10^–3^	2.8 × 10^–3^	1.8 × 10^–3^	52.8 × 10^–3^

Experimental conditions: 0.58 mg NADH. CO_2_ flow rate equals 100 cm^3^/min, reaction time equals 5 h. Volume of PB = 1.41 mL

On the contrary, FA concentration obtained for soluble cbFDH showed a linear increasing behaviour with the protein-to-NADH ratio (expressed as mg_cbFDH_ /µmol_NADH_) with almost negligible amount of FA (2.8 microM) for a protein-to-NADH ratio of 0.43 (Figure ESI-7). This is remarkably much lower than the value (261 microM) obtained for a protein-to-NADH ratio of 0.56 when cbFDH is immobilized in G200 carbon. This remarkable difference in the concentration of FA could be associated to the vigorous bubbling of CO_2_ through the protein solution, which seems to be detrimental for the stability of cbFDH [31] prompting to rapid inactivation compared to immobilization.

Following chemical reaction (3), the confined cbFDH provides FA yields between 45 and 36% for the carbons with cBFDH to NADH ratios between 0.024 (G200K) and 0.66 (G200CB), whereas the FA yield using soluble cbFDH is 28% with a cbFDH-to-NADH ratio of 18 after 5 h of reaction. It should be mentioned that herein reported yield values of FA formation are still far from those reported by Fernandez-Lafuente et al. [48] for both soluble cbFDH (80% in bicarbonate buffer solution) and cbFDH immobilised in polyvinyl alcohol polymeric matrix (92%) after 4 h of reaction. However, our study shows the viability of mesoporous carbons as porous matrices for the immobilization of cbFDH. Accordingly, the reaction time could increase the concentration of FA up to a maximum value, since NADH species are the limiting reagents: starting amount of 0.82 micromol compared to the ca. 46 micromol of CO_2_, according to solubility data [49], during the whole reaction.

3 $${\text{CO}}_{2} + 2{\text{NADH}}\overset {{\text{cbFDH}}} \longleftrightarrow {\text{HCOOH}} + 2{\text{NAD}}^{ + }$$

However, it should be noted that we observed a gradual adsorption of NADH in the porosity of the carbons during the 5 h of reactions (according to absorbance decrease at 340 nm), following the trend: G100 > G200 > G200CB > G200K (Figure ESI-8). It is worth noting that for sample G200K, NADH adsorption is low (complete depletion is not achieved), for which an increase in the reaction time would have probably increased the FA concentration yield. Simultaneously to NADH adsorption, we have observed a gradual oxidation of NADH to NAD^+^, and further adsorption of the later (see Figure ESI-9), catalyzed by all the carbon materials examined in this study i.e., absorbance at 259 nm increases upon decreasing the absorbance band at 350 nm mainly associated to NADH; this behavior has been also reported by other carbon materials potentials [50]. The above behavior is also supported by the lack of oxidation of NADH in PB solution in the absence of the carbons (see Figure ESI-10).

As far as the production rate is concerned, when the FA production rate is normalized by the amount of cbFDH (i.e., µmol FA/min mg, Table 5), a remarkable improvement in the FA synthesis rate is obtained for all the mesoporous carbons, at least more than one order of magnitude. This enhancement indicates the more stable conformation and higher biocatalytic response of the immobilized enzyme compared to the enzyme in solution under our experimental conditions of bubbling CO_2_.

Even though the FA production rate depends on the cbFDH concentration in solution under an excess of NADH, only cbFDH-to-NADH ratios between 0.2 and 0.7 are beneficial for a high FA production of two orders of magnitude (samples G100, G200 and G200CB). This fact could be explained by a proper enzyme confinement trade-off on the mesoporous carbon with retention of the conformational structure and enzymatic activity. It is interesting to remark the performance of sample G200K, leading to an amount of FA similar to that of the enzyme immobilized in G100 and G200, despite the low loading of cbFDH of the former (Table 5). The normalized production in terms of µmol FA/mg cbFDH min is thus much higher in G200K, although the production rate in terms of µmol FA/min is quite similar for all the systems.

The correlation between the catalytic activity of immobilized cbFDH and the average mesopore size of the carbons is also shown in Figure ESI-11. The samples showing the higher FA production rates are those which main mesopore cavities match the dimensions of the enzyme—provided that the pore mouths are not too small to hinder the accessibility of the enzyme inside the pores—to favor a tight pore confinement (e.g., G200K and G100). In the case of sample G200CB, despite its higher amount adsorbed and higher conductivity, the normalized FA production rate (considering the amount of enzyme immobilized) is the lowest of the series, corroborating the critical role of the confinement (over conductivity). However, some questions do still arise from the above discussion since FA production depending on amount of immobilized cbFDH with time-on-stream should be performed for a better elucidation and understanding of the effect of main cavity and pore mouth dimensions and therefore, to obtain the optimized cbFDH concentration per mg of carbon material. Thus, further experiments must be performed to elucidate the role of the pore necks dimensions in sample G200K as the step determining process accounting for a presumably partial confinement of the protein which would provide a more efficient accessibility of NADH and CO_2_.

Although further evaluation of the enzymatic activity mechanisms through a deep analysis of kinetic parameters (e.g., Michaelis constant, turnover number) would provide essential information for understanding the effect of the textural properties on the activity of the enzyme, and would allow an adequate comparison of the FA production rates, our study demonstrates the viability of the mesoporous carbons as supports for improving the stability and activity of cbFDH for the production of formic acid, and points out the importance of matching the dimensions of the enzyme and the pores of the host material for an improved catalytic response.

## Conclusion

The nanoconfinement of Cyt c and cbFDH on carbons with specially designed mesoporosity has revealed itself as an important breakthrough in the development of biosensors for detecting hydrogen peroxide and in the preparation of long-term stable porous materials as supports for the enzymatic reduction of CO_2_ to formic acid. The immobilization of both studied biocatalysts on the selected mesoporous carbons has been found to be strongly dependent on both the dimensions of the pore cavities and the pore necks in terms of overall uptake. Regarding the electrocatalytic activity, the highest sensitivity and lowest LoD were obtained for Cyt c immobilized in the carbons combining adequate dimensions of the main mesopore cavities and accessibility through big enough pore neck, with evidences of direct electron transfer when the dimensions of Cyt c and the pores matched. While the electrical conductivity seems to affect the sensitivity of the electrodes (e.g., controlling current losses), the LoD is governed by the optimal confinement of the biomolecules in the porosity of the carbon materials. On the other hand, the activity of the nanoconfined cbFDH enzyme on the selected mesoporous carbons was also examined towards formic acid production. A sluggish immobilization was observed on the carbons exhibiting the smallest pore neck dimensions (ca. G100 and G200K), demonstrating the key role of the accessibility of the porous network. The nanoconfinement of cbFDH resulted in a considerable increase in its catalytic activity, being the production rate of formic acid much higher for the enzyme immobilized in the carbons than in solution. Overall, higher FA production rates were obtained in those carbon samples which pore structure is large enough to accommodate the enzyme, provided that the pore necks are not too small to hinder the accessibility of the enzyme and the substrate.

## Supplementary Information

Below is the link to the electronic supplementary material.

Supplementary file1 (DOCX 7687 KB)
