# Supplementary material for: Effect of confinement of horse heart cytochrome c and formate dehydrogenase from Candida boidinii on mesoporous carbons on their catalytic activity
Source: Bioprocess Biosyst Eng. 2021 Apr 3;44(8):1699–710. doi: 10.1007/s00449-021-02553-3 (PMC8238777; doi:10.1007/s00449-021-02553-3)
Supplement: Supplementary file 1 — Supplementary file1 (DOCX 7687 KB) [file 449_2021_2553_MOESM1_ESM.docx]

**Supplementary information**

**Effect of confinement of horse heart cytochrome c and formate dehydrogenase from *Candida boidinii* on mesoporous carbons on their catalytic activity**

Naiara Hernández-Ibáñez^1^, Vicente Montiel^1^, Alicia Gomis- Berenguer^3^, Conchi Ania^2,3*^, and Jesús Iniesta^1^*

*^1^ Physical Chemistry Dept. and Institute of Electrochemistry, University of Alicante, 03080 Alicante, Spain.*

*^2^ INCAR, CSIC, Apdo 26, 33011 Oviedo, Spain*

*^2^ CEMHTI, CNRS (UPR 3079) University of Orléans, 45071, Orléans, France*

Corresponding authors: [jesus.iniesta@ua.es](mailto:jesus.iniesta@ua.es) and conchi.ania@cnrs-orleans.fr

**Table ESI-1.** Main textural parameters of the mesoporous carbons obtained from the equilibrium N_2_ adsorption/desorption isotherms at ‑196 ºC.

|  | G100 | G200 | G200CB | G200K |
| --- | --- | --- | --- | --- |
| **S_BET_ [**m^2^ g^-1^**]** | 867 | 832 | 653 | 1262 |
| **V_TOTAL PORES_ ^A^** [cm^3^ g^‑1^] | 1.15 | 1.47 | 1.26 | 0.81 |
| **V_MICROPORES_ ^B^** [cm^3^ g^‑1^] | 0.16 | 0.17 | 0.10 | 0.46 |
| **V_MESOPORES_** ^B^ [cm^3^ g^‑1^] | 0.89 | 1.23 | 1.08 | 0.34 |
| **V_MESOPORES_/** V_TOTAL PORES_ [%] | 77 | 84 | 86 | 42 |
| ^A^ Total pore volume evaluated at p/p_0_ ~0.99 | | | | |
| ^B^ micropore/mesopore volumes evaluated applying the two dimensional nonlinear density functional theory model (2D-NLDFT) assuming surface heterogeneity (SH) of pores. | | | | |

Samples G100 and G200 displayed similar surface areas and micropore volumes, whereas sample G200CB displayed a slightly lower surface area and micropore volume. However, the chemically activated carbon (sample G200K) presented higher surface area and microporosity, although the total pore volume and mesopore volumes (i.e., mesopore fraction) were the lowest of the series. This is due to the development of the microporosity upon the activation of the sample.

**Figure ESI-1**. SEM images of selected mesoporous carbons showing the average particle sizes after sieving for the preparation of the electrodes.

**Figure ESI-2.** High resolution equilibrium N_2_ adsorption/desorption isotherms at ‑196 ºC of the studied mesoporous carbons.

All the carbons display type IVa isotherms [[1](#_ENREF_1)], with prominent hysteresis loops in the desorption branch, characteristic of micro/mesoporous materials. Interesting differences can be observed in the pore volumes and the position of the hysteresis loops. For instance, samples G100, G200 and G200CB present hysteresis loops with somewhat parallel adsorption/desorption branches, associated with the presence of uniform (meso)pores size distributions [[1](#_ENREF_1)]. Additionally, the loops shift towards higher relatives pressures, indicating larger mesopores following the trend: G100<G200<G200CB. At converse, carbon G200K shows a type H2(a) loop with a steep desorption at p/p_0_ ~ 0.5. This is characteristic of materials with pore constrictions [[2](#_ENREF_2)].

**Figure ESI-3.** Pore size distributions of the studied carbons obtained from the nitrogen isotherms at -196 ºC by applying the 2D-NLDFT-HS model to the (A) adsorption and (B) desorption branch.

Pore size distribution profiles have been obtained by the analysis of both the adsorption and desorption branches of the N_2_ adsorption/desorption isotherms. This is recommended in the literature for a realistic characterization of complex pore systems where percolation effects are suspected [[2](#_ENREF_2)], as it is the case of herein studied materials. In such cases, and provided that there are no cavitation effects, the analysis of the adsorption branch of the N_2_ isotherm provides information about the size of main mesopore cavities (e.g., pore bodies) while the analysis of the desorption branch gathers information about the size of the pore necks.

**Figure ESI-4.** Chronoamperometric response at -0.5 V for the electroreduction of H_2_O_2_ using G200/GCE and Cyt-c/G200/GCE over consecutive additions of H_2_O_2_ solution in 0.1 M PB pH 4.0. Arrows indicate the addition of H_2_O_2_.

|  |  |
| --- | --- |
|  |  |

**Figure ESI-5.** Correlations between the electrocatalytic parameters -sensitivity for the detection of H_2_O_2_ (A, C) and limit of detection (B, D)- as a function of the mesopore dimensions (main cavity and pore mouths) at pH 7.0 (A,B) and 4.0 (C, D).

**Figure ESI-6.** Production rates of formic acid (FA) expressed in µmol FA min^-1^ (black squares) and in µmol FA min^-1^ mg^-1^_cbFDH_, (red squares) using the cbFDH in aqueous solution as a function of .protein to NADH ratio (mg_cbFDH_ / µmol_NADH_.: cbFDH protein dissolved in 0.1 M PB pH 7.4, 0.58 mg NADH. CO_2_ flow rate equals 100 cm^3^ min^-1^; reaction time equals 5 h. Volume of PB solution = 1.41 mL.

**Figure ESI-7.** Formic acid concentration as a function of protein to NADH ratio (mg_cbFDH_ / µmol_NADH_.: cbFDH protein dissolved in 0.1 M PB pH 7.4, 0.58 mg NADH. CO_2_ flow rate equals 100 cm^3^ min^-1^; reaction time equals 5 h. Volume of PB solution = 1.41 mL.

**Figure ESI-8.** UV-vis absorbance of the supernatant NADH solutions after 5 hours of NADH adsorption on the porous carbons. All the carbons were dispersed in 10 mM PB pH 7.4 to reach a final 1.0 mg mL^-1^ ink concentration and then stirred at room temperature for 24 hours. Then, 205 microliters of an initial NADH solution with a concentration of ca. 1.0 mg/mL in 10 mM PB (pH 7.4) was added into the carbonaceous ink before NADH adsorption started. Adsorption experiments was performed under inert atmosphere. All the solutions were diluted using a 10 mM Pb pH 7.4 solution with a dilution factor of 5.88.

**Figure ESI-9** UV-vis absorbance of the supernatant NAD^+^ solutions after 5 hours of NADH adsorption on the porous carbons. All the carbons were dispersed in 10 mM PB pH 7.4 to reach a final 1.0 mg mL^-1^ ink concentration and then stirred at room temperature for 24 hours. Then, 205 microliters of an initial NAD^+^ solution with a concentration of ca. 1.0 mg/mL in 10 mM PB (pH 7.4) was added into the carbonaceous ink before NAD^+^ adsorption started. Adsorption experiments was performed under inert atmosphere. All the solutions were diluted using a 10 mM Pb pH 7.4 solution with a dilution factor of 5.88.

**Figure ESI-10.** UV-vis absorbance of NADH solutions with time to evidence the stability of the solution. 205 microliters of an initial NADH solution with a concentration of ca. 1.0 mg/mL in 10 mM Pb pH 7.4) was added into 1.0 mL 10 mM PB pH 7.4. Experiments were performed under inert atmosphere.

**Figure ESI-11.** Correlation between the FA production rate and the mesopore dimensions (main cavity and pore mouths).

___________________________________

**References**

[1] M. Thommes, K. Kaneko, A.V. Neimark, J.P. Olivier, F. Rodriguez-Reinoso, J. Rouquerol, K.S.W. Sing, Pure and Applied Chemistry 87 (2015).

[2] M. Thommes, K.A. Cychosz, Adsorpt 20 (2014) 233.
